# Case Report: Mucoepidermoid carcinoma in rare locations: a report of two cases

**DOI:** 10.3389/fonc.2025.1522968

**Published:** 2025-06-19

**Authors:** Chao Zhang, Baoqin Lu, Haoyu Shi, Zihan Ni, Yuyan Cao

**Affiliations:** ^1^ Department of Thyroid and Breast Surgery, Jiujiang University Affiliated Hospital, Jiujiang, China; ^2^ Department of Head and Neck Surgery, Jiangxi Cancer Hospital, The Second Affiliated Hospital of Nanchang Medical College, Nanchan, China

**Keywords:** mucoepidermoid carcinoma, lung, breast, histopathology, immunohistochemistry

## Abstract

Mucoepidermoid carcinoma (MEC) is a common malignancy of the salivary glands, accounting for 10-15% of salivary gland tumors. It mostly occurs in the salivary glands and very rarely in the lungs and mammary glands. The incidence of breast MEC is only 0.2% to 0.3% of all primary breast tumors. Pulmonary MEC accounts for only 0.1-0.2% of all lung tumors. This report presents two cases of MEC occurring in rare locations: the left lower lung and the breast. Both cases presented different clinical features and histopathological characteristics. The immunohistochemical analysis of Ki-67, CK5/6, P63 and other positive results also confirmed the diagnosis. Surgical resection was the primary treatment in both cases, with the patient with lung involvement additionally receiving chemotherapy and radiotherapy. These cases underscore the importance of recognizing MEC in atypical locations, as timely diagnosis and proper treatment approaches are vital for improving patient outcomes. From these cases, the complexity of MEC is highlighted and the necessity of precise histopathological diagnosis, especially in rare sites, is emphasized.

## Introduction

Mucoepidermoid carcinoma (MEC) is a malignant tumor primarily found in the salivary glands, accounting for 10–15% of all salivary gland tumors and 30% of all salivary gland malignancies (1), with a higher prevalence in women (51.5%) (2). The parotid gland (56.8%) and the hard palate (18%) are typical sites of involvement (3). While MEC is more commonly found in the salivary glands, it can also occur in extra-salivary tissues, such as the lung, pancreas, thyroid, accessory lacrimal glands, and breast, though these occurrences are rare (4–8). MEC of the breast is an exceedingly rare malignancy, constituting only 0.2–0.3% of all breast cancers (9). The first case of breast MEC was reported by Patchefsky et al. in 1979 (10). By 2022, only 45 cases of breast MEC had been documented worldwide, with only four cases classified as intermediate-grade MEC. Pulmonary MEC is a subtype of non-small cell lung cancer (NSCLC) and is a small proportion (0.1–0.2%) of primary lung cancers and primarily affects younger individuals (10).

Histologically, MEC is characterized by a combination of squamous cells, mucinous cells and intermediate cells. Based on histological features, MEC is classified into low-grade, high-grade subtype and intermediate type. Low-grade MEC predominantly consists of glandular elements and mucinous cells, whereas high-grade MEC contains mostly squamous and intermediate cells, with fewer mucinous cells. Low-grade MEC is usually associated with a good prognosis, with a 5-year survival rate of 90-100% (1), while high-grade MEC is associated with a poorer prognosis. The intermediate type is somewhere in between. MEC occurring in atypical sites presents unique diagnostic challenges. These rare presentations can be misdiagnosed as more common malignancies, complicating clinical management. Accurate diagnosis in such cases relies on histopathological and immunohistochemical analysis (11).

This report adds to the growing body of knowledge regarding MEC in non-salivary gland locations and emphasizes the importance of recognizing atypical presentations to ensure accurate diagnosis and appropriate treatment (8). While most MEC cases involve the salivary glands, particularly the parotid gland, this report of MEC in a rare site underscores the need to better understand the clinical behavior, prognosis, and treatment strategies for MEC outside of traditional anatomical locations (12).

## Case 1: MEC of the breast

**Figure 1:**
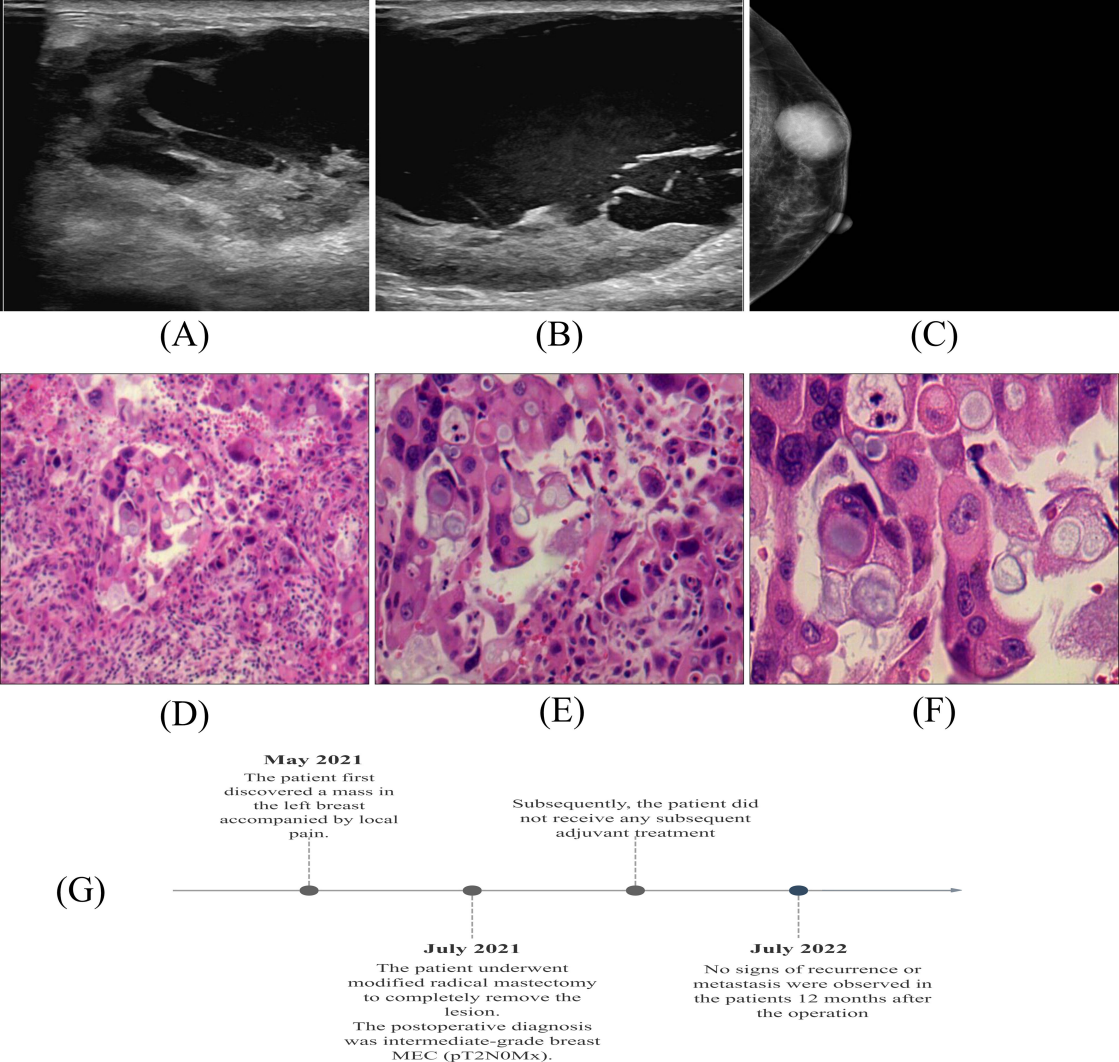
Imaging of the breast: **(A, B)** Ultrasonography. **(C)** Mammography. Histology of breast MEC: **(D)** ×100; **(E)** ×200; **(F)** ×400. **(G)** Patient Treatment Schedule.

A 47-year-old female came to the hospital with a left breast mass with local pain 2 months ago. During physical examination, a mass of about 5.0cm×4.0cm in size was palpated near the edge of the breast at 2 o’clock in the upper outer quadrant of the left breast. The mass was soft, regular in shape, well bounded, with fair movement and tenderness. No obvious mass was palpable on the right breast. No obvious nipple discharge or other abnormalities were observed. Enlarged lymph node of the size 2.0×1.0cm was palpated in the left axilla, while no enlarged lymph nodes were palpated in the right axilla. In addition, the patient had no family history of breast cancer. The ultrasound examination of the breast reported that at 2 o’clock on the left breast of the patient, there was a 4.65 × 4.52 × 2.94cm mixed cystic and solid echo mass on the edge of the entry line, with a clear boundary, a long oval shape and irregular thickening of the wall. In addition, there were crisscrossed strip diaphragm echo-mass, with a low to no echo-zone in the center, and the aspect ratio was less than 1. At 2 o’clock on the right breast, a 0.61 × 0.23cm solid hypoechoic area can be seen at 2 cm from the nipple, the boundary is not clear, it is elongated, and the aspect ratio is less than 1. Several hypoechoic nodules of different sizes were seen in the left axilla, the larger one was about 2.26×0.9cm, and the central medullary structure was slightly reduced and slightly eccentric. No abnormal enlarged lymph nodes were observed in the right axilla (**Figures 1A-C**). Subsequently, the patient underwent a modified radical mastectomy to remove the lesion completely. According to the postoperative pathological and immunohistochemical results, the patient was diagnosed with intermediate-grade MEC. After multidisciplinary discussions, it was considered that the benefit of adjuvant chemotherapy for this patient was unclear, and radiotherapy was recommended. However, the patient did not receive any subsequent adjuvant therapy in the end. Twelve months after the operation, the patient was in good health with no signs of recurrence or metastasis. Furthermore, we developed a comprehensive patient treatment timeline that systematically delineates the therapeutic interventions and clinical progression (**Figure 1G**).

The gross examination of the resected tumor showed that the lesions were mainly solid and clearly defined, with gray white and gray red sections, with a volume of 5×3.4×2cm. Under the microscope, the tumor is composed of a large number of atypically proliferating intermediate cells, a small number of squamous cells, and a small number of mucinous cells. The tumor cells are arranged in solid or glandular patterns, with focal necrosis visible. Mitotic figures are easily seen, and neural invasion is present. (**Figures 1D-F**).

**Figure 2:**
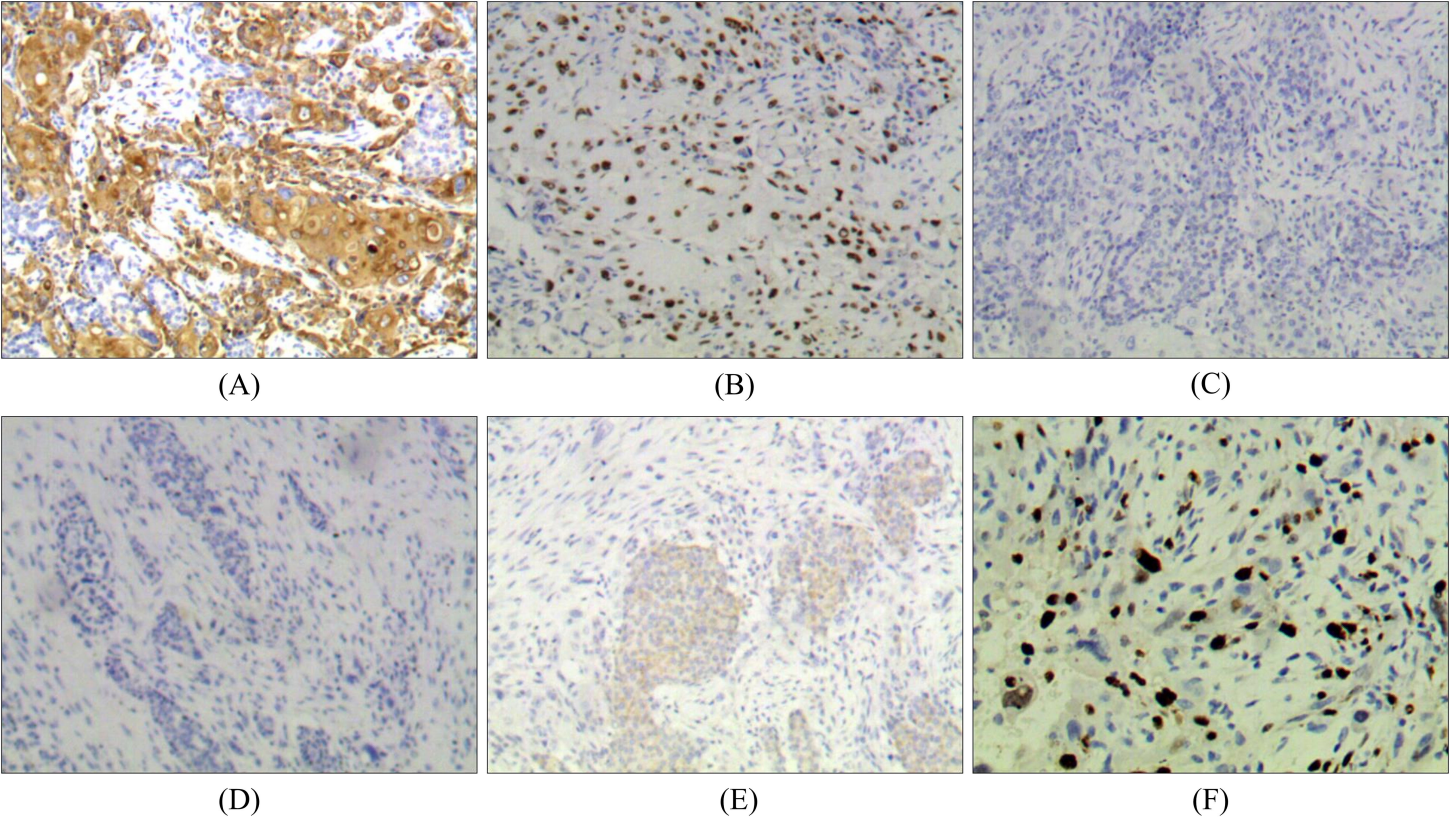
Immunohistochemistry of breast MEC: **(A)** Positive for CK5/6 (×100). **(B)** Positive for p63 (×100). **(C)** Negative for ER (×100). **(D)** Negative for PR (×100). **(E)** Negative for HER-2 (×100). **(F)** Positive for Ki67 (+, 30%).

Immunohistochemical results showed that tumor cells expressed CK5/6, p63 positive. Tumor cells did not express positive estrogen receptor (ER), progesterone receptor (PR) and HER-2/NEU protein. About 30% of tumor cells were positive for Ki67. Combined with histopathological findings, this case was classified as intermediate-grade breast MEC (**Figure 2**).

## Case 2: MEC of the lung

**Figure 3:**
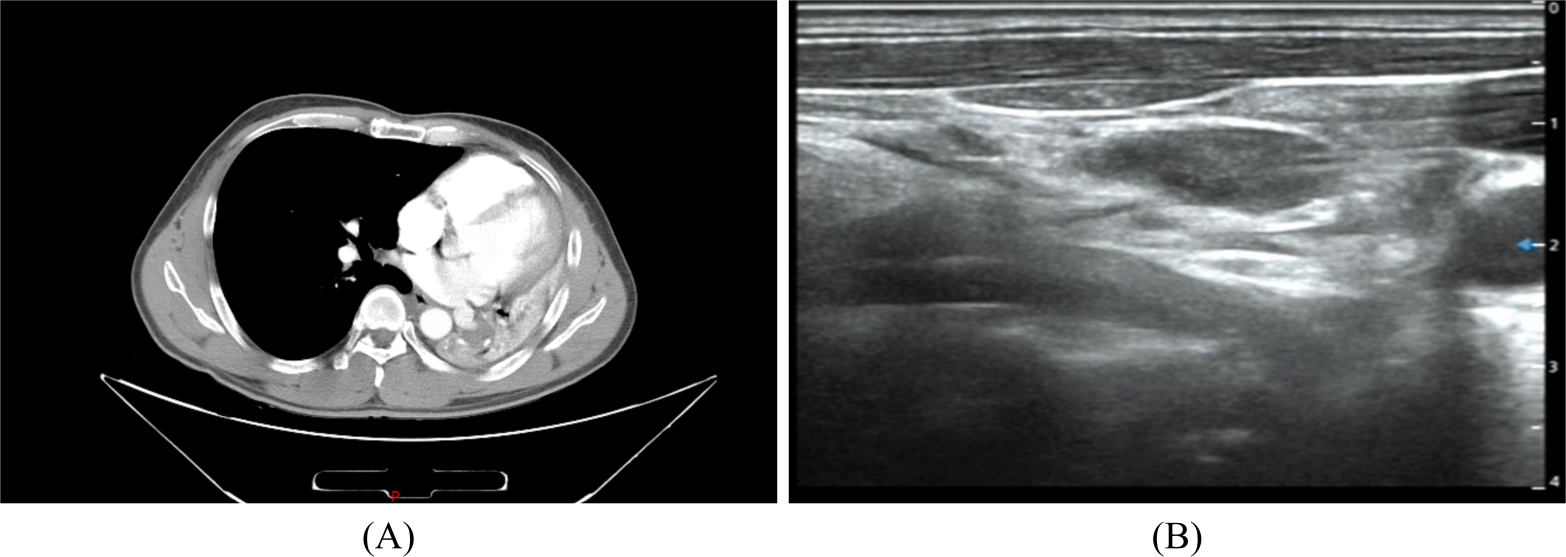
Chest CT **(A)** The left lung is reduced in volume and multiple nodules are seen in the lower left lung. Neck ultrasound **(B)** Multiple swollen lymph nodes in the neck.

**Figure 4:**
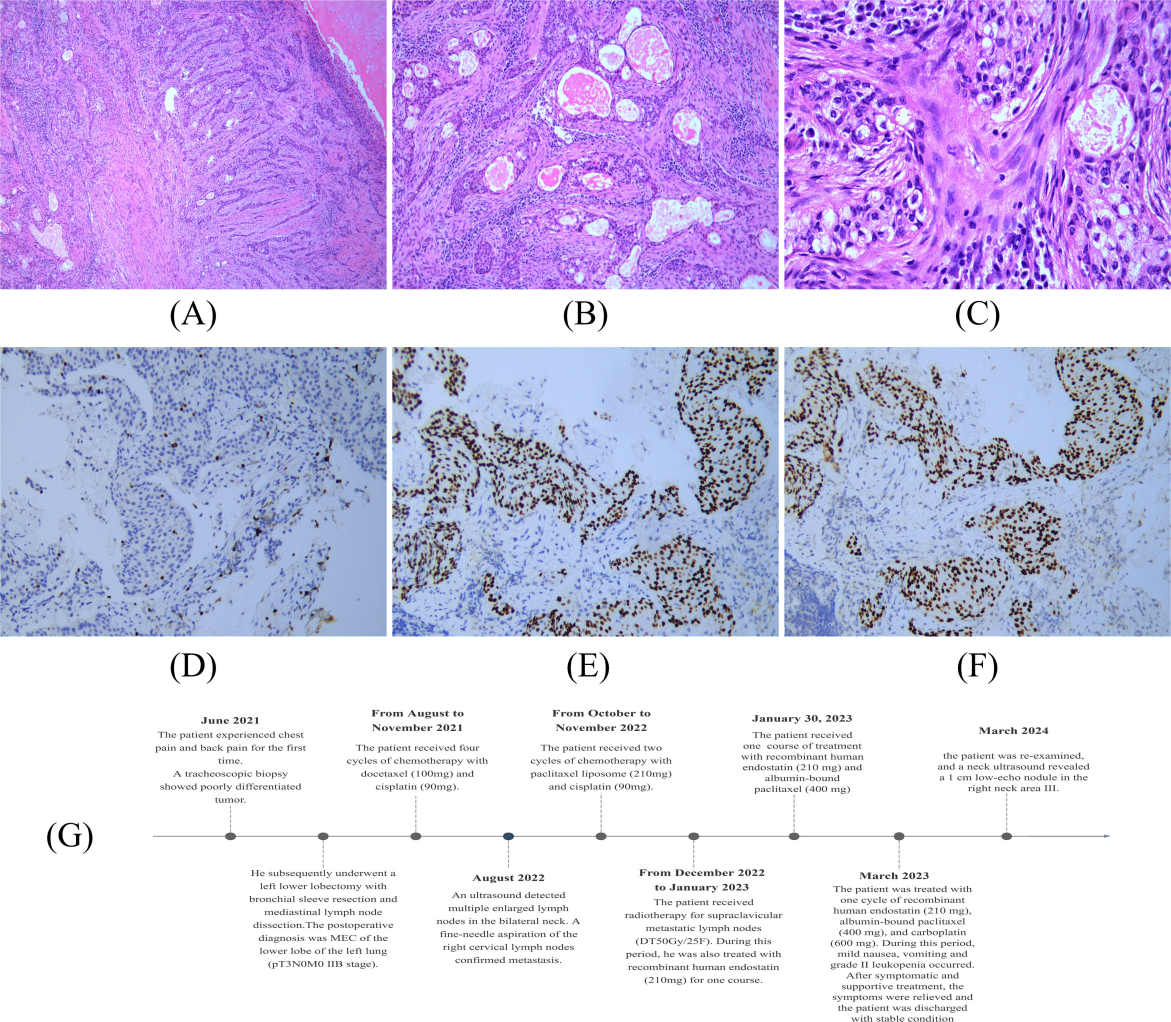
Histopathological examination of pulmonary MEC: **(A)** ×100; **(B)** ×200; **(C)** ×400. Immunohistochemistry of Pulmonary MEC: **(D)** Positive for Ki67 (+, 10%). **(E)** Positive for P40 (×200). **(F)** Positive for P63 (×200). **(G)** Patient Treatment Schedule.

A 45-year-old male was admitted to the hospital for recurrence of MEC of the left lower lung more than one month after his last treatment. The patient initially visited the hospital in June 2021 because of chest and back pain, with a chest CT scan revealing several slightly weak and enhanced nodules in the left lower lobe, the largest being 2.2 cm × 1.4 cm, presenting as an irregular solid shadow (**Figure 3A**). A tracheoscopic biopsy revealed a poorly differentiated tumor. He subsequently underwent a left lower lobectomy with bronchial sleeve resection and mediastinal lymph node dissection. Intraoperative freezing showed negative bronchial margins and lymph nodes. Postoperative pathology confirmed MEC invading the cartilage. Histopathological examination with hematoxylin and eosin (HE) staining revealed a mixture of squamous cells, intermediate cells, and mucinous cells. The glandular structure containing mucin is obvious, with moderate nuclear pleomorphism and some hyperchromic nuclei (**Figures 4A-C**). Immunohistochemistry was positive for Ki-67 (+, 10%), CK5/6(+), CK7(+), P63(+), and p40(+) (**Figures 4D-F**), consistent with MEC. The patient was diagnosed postoperatively with stage IIB (pT3N0M0) disease and received four cycles of chemotherapy with docetaxel and cisplatin from August to November 2021.

In August 2022, an ultrasound detected multiple enlarged lymph nodes in the bilateral neck (**Figure 3B**). A fine-needle aspiration of the right cervical lymph nodes confirmed metastasis. From October to November 2022, he received two additional cycles of chemotherapy with paclitaxel liposome and cisplatin and then received radiotherapy for supraclavicular metastatic lymph nodes (DT50Gy/25F) between December 2022 and January 2023. During this period, he was also treated with recombinant human endostatin. In March 2023, the patient was admitted for further treatment. The patient had a history of smoking for more than 20 years, 20 cigarettes a day, and had quit smoking for one month. Physical examination revealed an old surgical scar on the chest wall, with clear lung sounds and no palpable lymphadenopathy. Auxiliary examinations showed no obvious abnormalities on brain MRI and CT scans of the chest, and upper abdomen, which indicated no significant changes compared to a previous scan. The Neck CT showed multiple small lymph nodes. The bone scan showed benign changes without evidence of bone metastasis.

The patient was treated with one cycle of recombinant human endostatin (210 mg), albumin-bound paclitaxel (400 mg), and carboplatin (600 mg) starting on March 16. During hospitalization, he developed mild nausea, vomiting, and grade II leukopenia, but these symptoms were managed with supportive care. He was discharged in a stable condition with a diagnosis of maintenance chemotherapy for left lower lobe MEC (rT0N3M0). In March 2024, the patient was re-examined, and a neck ultrasound revealed a 1 cm low-echo nodule in the right neck area III. The prognosis remains guarded given the aggressive nature and recurrent behavior of the tumor. Similarly, we have formulated a comprehensive patient treatment schedule in order to improve the readability of the report (**Figure 4G**).

## Discussion

Case reports of MEC at rare sites highlight the complexity and aggressiveness of this rare malignancy. In the context of relevant literature, we discussed and summarized the rare occurrence sites, treatment methods and metastasis of the disease (**Table 1**). The aim is to provide clinicians with valuable insights into the diagnosis and management of this rare disease.

**Table 1:** Summary of case reports on MEC in rare sites.

Author	Year	Age	Sex	Diagnosis	Organism	Metastasis	Treatment
Yuan Chen et al. (54)	2024	51	Female	Pancreatic mucoepidermoid carcinoma	Pancreas	Yes	Pancreaticoduodenectomy and chemotherapy
Márcio Rodrigues Costa et al. (55)	2015	47	Male	Mucoepidermoid carcinoma of the penis	Penis	Yes	Total penectomy and chemotherapy
Himsikhar Khataniar et al. (56)	2022	51	Female	Mucoepidermoid carcinoma of the anterior mandible	Anterior mandible	No	Segmental mandibulectomy with reconstruction
Janakiram T. N. et al. (57)	2016	65	Male	Primary mucoepidermoid carcinoma of the lacrimal sac	Lacrimal sac	Unknown	Radical surgery and chemoradiation
Zhang HY and Yang HY (58)	2020	39	Female	Mucoepidermoid carcinoma in the infratemporal fossa	Infratemporal fossa	Yes	Extended resection of primary tumor, radical neck dissection and radiotherapy
Mario Della Mura et al. (59)	2023	58	Female	High-grade HER2-positive breast mucoepidermoid carcinoma	Breast	No	Surgical resection, chemotherapy and targeted therapy
Alessandro G. Fois et al. (60)	2017	46	Male	Mucoepidermoid carcinoma of the bronchus	Bronchus	No	Left upper lobectomy and mediastinal lymphadenectomy
Zihan Li et al. (61)	2024	67	Male	Primary hepatobiliary mucoepidermoid carcinoma	Liver	Unknown	Surgical resection
Manabu Yamamoto et al. (62)	2018	74	Male	High-grade mucoepidermoid carcinoma of the anal canal	Anal canal	Yes	Local surgical resection and irradiation​ therapy

MEC is a rare epithelial malignant tumor, usually presenting in salivary glands and was first discovered by Foote et al (13). MEC show a strong preference for the parotid gland, most commonly with low grade histology. MEC outside the salivary glands is very rare. Breast MEC is particularly rare. At present, only 45 cases of breast MEC have been reported in English literature. All patients were female, aged 27–86 years old. There were 19 cases of severe MEC, 20 cases of low MEC, 4 cases of moderate MEC, and the remaining 2 cases were not explained. **Table 2** summarizes the references and publication years of these studies, as well as the clinicopathological features of these 45 patients and current cases. Pulmonary MEC is a malignant tumor originating in the bronchial glands, which was first described by Smetana in 1952 (3), with a presumed incidence of 0.1-0.2% of all lung cancers (14). Given the rarity of the disease, especially in non-salivary gland sites, MEC often has non-specific clinical features, making the diagnosis of MEC in these atypical sites challenging and often easily misdiagnosed as other common diseases at that site. For example, MECS with large cystic structure are easily mistaken for single breast cyst, while MECS with microcystic structure are easily mistaken for ductal carcinoma *in situ*. In this case, the breast MEC showed features associated with generally benign breast lesions, which guided our preoperative discussions with the patient and their family, focusing on a more favorable prognosis. However, the final diagnosis of MEC, a rare malignancy, presented unexpected challenges. This underscores the need for better vigilance in similar cases. In the future, even when a tumor appears benign preoperatively, clinicians must maintain a differential diagnosis that includes the possibility of malignancy, especially in cases involving atypical presentations or rare histological subtypes. The implications of underestimating such cases could lead to delays in appropriate treatment and affect patient outcomes. Therefore, preoperative planning should thoroughly consider the possibility for malignancy to ensure timely, accurate diagnosis and treatment.

**Table 2:** Summary of reported cases of breast MEC from 1979 to 2024.

Study	Year	Age	Site	Size	Grade	LN	Distant	Follow-up	Status
		(Years)		(cm)		Metastasis	Metastasis	(Months)	
Present study	2024	48	Left	5	IG	0/4	No	12	Alive
Wang et al (27)	2024	47	Left	4.5	HG	No	No	12	Alive
Mura et al (28)	2023	58	Left	2	HG	No	No	61	Alive
Bak et al (29)	2022	47	Right	3.2	IG	No	No	37	Alive
Ye Ru-Pei et al (30)	2020	42	Right	2.6	LG	NA	No	12	Alive
Mingfei Yan et al (31)	2019	60	Right	1.9	LG	NA	No	60	Alive
Burghel et al (32)	2018	73	Left	NA	LG	0/2	No	NA	NA
Sherwell-Cabello et al (7)	2017	86	Left	6	LG	NA	No	3	Alive
Cheng et al (33)	2017	39	Right	1.5	LG	3/18	No	156	Alive
		49	Left	1.5	LG	0/17	No	41	Alive
		66	Left	1.3	LG	0/6	No	9	Alive
		61	Left	3	LG	0/3	No	4	Alive
Fujino et al (34)	2016	71	Right	1.7	IG	0/NA	No	NA	NA
Palermo et al (35)	2013	80	Right	4	HG	0/NA	No	NA	NA
Turk et al (36)	2013	40	Right	5.5	NA	1/24	No	5	Alive
Basbug et al (21)	2011	69	Left	10	HG	0/12	No	12	Alive
Camelo-Piragua et al (37)	2009	49	Right	4	IG	1/3	No	8	Alive
Hornychova et al (38)	2007	62	Right	1.8	HG	0/17	No	18	Alive
		30	Left	8	LG	0/NA	No	60	Alive
Horii et al (16)	2006	54	Left	2.5	LG	0/NA	No	36	Alive
Gomez-Aracil et al (39)	2006	69	Right	7.5	HG	24/28	No	54	Alive
Di Tommaso et al (40)	2004	80	Left	0.5	LG	NA	No	5	Alive
		29	Left	0.8	LG	NA	No	90	Alive
		54	Left	1.5	LG	NA	No	13	Alive
		55	Left	1.1	IG	NA	No	18	Alive
		36	Left	0.6	HG	NA	No	3	Alive
Terzi et al (41)	2004	79	Right	8	HG	4/14	NA	NA	NA
Tjalma et al (42)	2002	58	Right	3.5	HG	1/17	Yes	156	Alive
Berry et al (43)	1998	51	Left	3.5	HG	0/NA	No	NA	NA
Markopoulos et al (44)	1998	40	Right	2	HG	0/NA	No	60	Alive
Chang et al (45)	1998	54	Left	4.5	HG	0/9	No	48	Alive
Luchtrath and Moll (46)	1989	60	NA	5	HG	12/18	Yes	30	DOD
Pettinato et al (47)	1989	72	Right	7	HG	16/19	Yes	10	DOD
Hanna and Kahn (48)	1985	51	Left	2	HG	0/NA	No	8	Alive
		31	NA	NA	NA	2/18	No	14	Alive
Hastrup and Sehested (49)	1985	59	Left	1	HG	0/4	Yes	25	DOD
Leong and Williams (50)	1985	57	Left	3.5	HG	0/20	Yes	7	DOD
Ratanarapee et al (51)	1983	27	NA	NA	HG	6/15	Yes	14	DOD
Fisher et al (52)	1983	65	Right	2	LG	NA	No	60	Alive
		71	Left	2	LG	0/19	No	48	Alive
		57	Right	2.5	LG	0/11	No	120	Alive
		49	Right	3.7	LG	0/13	No	108	Alive
		60	Left	4	LG	NA	No	48	DOR
Kovi et al (53)	1981	46	Left	11	HG	17/19	NA	NA	NA
Patchefsky et al (10)	1979	66	Right	1.3	LG	0/20	No	94	DOR
		70	Right	5	LG	NA	No	10	Alive

This case underscores the importance of distinguishing MEC from other tumor types through histopathological and immunohistochemical analyses, both of which are crucial for an accurate diagnosis (15). Histologically, MEC consists of mucinous cells, intermediate cells, and squamous cells. Based on the degree of cytological abnormalities and the relative proportions of these cell types, MEC can be classified into three grades: low, intermediate, and high (16). Low-grade MEC accounts for the majority of cases (48%), followed by high-grade (38.7%) and intermediate-grade (13.3%) (17). Histological grade is a key prognostic factor, with low-grade tumors exhibiting a higher proportion of mucinous cells and less aggressive behavior, while high-grade tumors contain fewer mucinous cells and have a worse prognosis (18). Intermediate-grade tumors display features that fall between these two extremes. In this study, the patient with breast MEC was pathologically diagnosed with intermediate-grade MEC. Tumor cells in breast MEC usually test negative for ER, PR, and HER-2/NEU protein (19), but breast MEC generally has a better prognosis than conventional triple-negative breast cancer (TNBC) (20). The immunohistochemistry results in this case aligned with these findings. Additionally, the tumor cells expressed p63, CK5/6, and epidermal growth factor receptor (EGFR), which is consistent with previously reported cases (21). Similarly, in Pulmonary MEC, the expression of Ki-67, CK5/6, p63, and p40 confirmed the diagnosis of MEC in this study. By successfully identifying MEC through thorough histopathological and immunohistochemical analysis, this case highlights the need for increased awareness among clinicians for such atypical presentations (22). This evidence adds value to future clinical practice by encouraging a multidisciplinary diagnostic approach, including the potential use of genetic markers for more precise diagnosis and treatment planning (23). Overall, this case strengthens the existing literature by emphasizing the importance of early detection and comprehensive diagnostic evaluations, which can significantly impact patient outcomes.

MEC is most effectively treated with surgery, with the extent of the procedure determined by the tumor’s location, size, and histopathological grade (18). Local resection is typically sufficient for low-grade, less aggressive tumors, whereas high-grade tumors require more extensive resections involving adjacent structures (18). Due to the rarity of breast MEC, research on this specific subtype is limited, and no standard treatment protocol exists. Consequently, breast MEC treatment is generally based on protocols used for more common breast cancers, with the surgical approach and postoperative treatment plan adjusted according to tumor location and grade. For low- and intermediate-grade breast MEC, the primary surgical options include modified radical mastectomy or mastectomy with sentinel lymph node biopsy. In contrast, high-grade breast MEC often requires more extensive procedures, such as modified radical mastectomy or radical mastectomy. Patients with high-grade MEC are recommended to receive postoperative adjuvant treatments including radiotherapy and chemotherapy to lower their chances of recurrence, and these patients should undergo closer monitoring with more frequent follow-ups. In the current case, the patient received a modified radical mastectomy, and postoperative histopathology confirmed intermediate-grade breast MEC. According to the characteristics of the tumor, the benefits of adjuvant chemotherapy were not clear. Radiotherapy was recommended, but in the end, the patient did not receive any subsequent adjuvant treatment. Twelve months after surgery, the patient remains in good health with no signs of recurrence. Surgical resection is also the standard treatment for pulmonary MEC. Low-grade pulmonary MEC generally has an excellent prognosis, with a 5-year survival rate of 95%, and adjuvant therapy is typically unnecessary. However, due to the limited number of high-grade pulmonary MEC cases, there is no established consensus about using adjuvant therapy in these patients. In the present case, the pulmonary MEC patient received a left lower lobectomy with bronchial sleeve resection and mediastinal lymph node dissection, and the surgical margins were negative. The tumor was histologically diagnosed as high-grade MEC. Despite postoperative chemotherapy, cervical lymph node metastasis occurred. Given that EGFR is often overexpressed in MEC of salivary gland origin, Han et al. identified EGFR mutations in 2 out of 5 Pulmonary MEC specimens (24). In this context, there have been several reports on the efficacy of the tyrosine kinase inhibitor gefitinib in patients with EGFR gene mutations (24, 25), and this molecular targeted therapy may improve outcomes for progressive high-grade and recurrent MEC cases. This case underscores the need for a multidisciplinary approach to diagnosis and treatment, especially in non-salivary gland cases, where the tumor’s clinical behavior and treatment options may vary from typical MEC cases (26). While surgical resection remains the primary treatment for MEC in most cases, the tumor’s grade and location can influence long-term outcomes and prognosis (12). Moreover, these two cases also highlight the importance of patient experience and compliance in the treatment of MEC in rare locations. For the breast MEC patient, attention to the breast lump and pain led to timely diagnosis and treatment of the disease. The Pulmonary MEC patient showed excellent treatment compliance. Despite having cervical lymph node metastasis, he actively cooperated with subsequent treatments.

## Conclusion

This case of MEC occurring in a rare anatomical location highlights the importance of considering MEC in differential diagnoses, even in atypical sites. The primary lesson learned from this report is the critical role of comprehensive histopathological evaluation in accurately diagnosing MEC, particularly when its clinical and radiological presentation overlaps with more common malignancies. The case reinforces the need for a multidisciplinary approach to ensure timely diagnosis and appropriate treatment. Early identification of the tumor’s grade and characteristics can significantly impact patient outcomes, because high-grade MEC requires more aggressive intervention. This report emphasizes the necessity for clinicians to maintain a high index of suspicion for MEC in unusual locations, as early detection and tailored treatment strategies can improve prognosis and overall patient care.

## Data Availability

The original contributions presented in the study are included in the article/supplementary material, further inquiries can be directed to the corresponding authors.

## References

[1] Peraza A Gómez R Beltran J Amarista FJ . Mucoepidermoid carcinoma. An update and review of the literature. J Stomatol Oral Maxillofac Surg. (2020) 121:713–20. doi: 10.1016/j.jormas.2020.06.003 32565266

[2] Limaiem F Lekkala MR Sharma S . "Mucoepidermoid lung tumor." In: StatPearls [Internet]. Treasure Island (FL): StatPearls Publishing. (2023). 30725962

[3] Smetana HF Iverson L L.L. SWAN . Bronchogenic carcinoma; an analysis of 100 autopsy cases. Military surgeon (1952) 111:335–51. 13002149

[4] Ma R Yu Y-Q Li J-T Peng S-Y . Mucoepidermoid carcinoma of the pancreas: a case report and a review of literature. J Res Med Sci. (2012) 17:886. 23826019 PMC3697217

[5] Sha AA La Fortune K Miller C Mills SE Baloch Z LiVolsi V . Thyroid sclerosing mucoepidermoid carcinoma with eosinophilia: a clinicopathologic and molecular analysis of a distinct entity. Modern Pathology. (2017) 30:329–39. doi: 10.1038/modpathol.2016.180 PMC5497311 27910944

[6] Dithmar S Wojno TH Washington C Grossniklaus HE . Mucoepidermoid carcinoma of an accessory lacrimal gland with orbital invasion. Ophthalmic Plast Reconstr Surg. (2000) 16:162–6. doi: 10.1097/00002341-200003000-00012 10749164

[7] Sherwell-Cabello S Maffuz-Aziz A Ríos-Luna NP Pozo-Romero M López-Jiménez PV Rodriguez-Cuevas S . Primary mucoepidermoid carcinoma of the breast. Breast J. (2017) 23(6):753–5. doi: 10.1111/tbj.12819 28397345

[8] Bai J Wang G . Mucoepidermoid carcinoma of the breast. Radiology. (2022) 305:32. doi: 10.1148/radiol.220128 35787204

[9] Bean GR Krings G Otis CN Solomon DA García JJ van Zante A . CRTC 1–MAML 2 fusion in mucoepidermoid carcinoma of the breast. Histopathology (2019) 74:463–73. doi: 10.1111/his.2019.74.issue-3 30380176

[10] Patchefsky AS Frauenhoffer CM Krall RA Cooper HS . Low-grade mucoepidermoid carcinoma of the breast. Arch Pathol Lab Med. (1979) 103:196–8. doi: 10.1097/00000478-197912000-00011 218522

[11] Mimica X Yuan A Hay A Katabi N Karassawa Zanoni D Valero C . Mucoepidermoid carcinoma: Evaluating the prognostic impact of primary tumor site. Oral Oncol. (2021) 123:105602. doi: 10.1016/j.oraloncology.2021.105602 34768210 PMC8671348

[12] Li Q Wei X Wang Y Liu C Fan B Lv C . Pulmonary mucoepidermoid carcinoma in the Chinese population: A clinical characteristic and prognostic analysis. Front Oncol. (2022) 12:916906. doi: 10.3389/fonc.2022.916906 36119481 PMC9476550

[13] Stewart FW Foote FW Becker WF . Muco-epidermoid tumors of salivary glands. Ann Surg. (1945) 122:820–44. doi: 10.1097/00000658-194511000-00005 PMC1618293 17858687

[14] Leonardi HK Jung-Legg Y Legg MA Neptune WB . Tracheobronchial mucoepidermoid carcinoma: clinicopathological features and results of treatment. J Thorac Cardiovasc Surg. (1978) 76:431–8. doi: 10.1016/S0022-5223(19)41067-2 703349

[15] Kimura M Miyajima K Ishikawa R Yamada Y Kono T Okunaka T . Photodynamic therapy for pulmonary mucoepidermoid carcinoma. Respir Med Case Rep. (2021) 33:101431. doi: 10.1016/j.rmcr.2021.101431 34401275 PMC8348553

[16] Horii R Akiyama F Ikenaga M Iwase T Sakamoto G . Muco-epidermoid carcinoma of the breast. Pathol Int. (2006) 56:549–53. doi: 10.1111/j.1440-1827.2006.02004.x 16930336

[17] Qureshi SM Janjua OS Janjua SM . Mucoepidermoid carcinoma: a clinico-pathological review of 75 cases. Int J Oral Maxillofac Pathol. (2012) 3:5–9. Available online at http://www.journalgateway.com.

[18] Kumar AN Nair PP Thomas S Raman PS Bhambal A . Mucoepidermoid carcinoma of sublingual gland: a Malignant neoplasm in an uncommon region. BMJ case reports (2011) 2011:bcr0220113864. doi: 10.1136/bcr.02.2011.3864 PMC3094775 22696723

[19] Jones C Ford E Gillett C Ryder K Merrett S Reis-Filho JS . Molecular cytogenetic identification of subgroups of grade III invasive ductal breast carcinomas with different clinical outcomes. Clin Cancer Res. (2004) 10:5988–97. doi: 10.1158/1078-0432.CCR-03-0731 15447982

[20] Pia-Foschini M Reis-Filho JS Eusebi V Lakhani SR . Salivary gland-like tumours of the breast: surgical and molecular pathology. J Clin Pathol. (2003) 56:497–506. doi: 10.1136/jcp.56.7.497 12835294 PMC1769991

[21] Basbug M Akbulut S Arikanoglu Z Sogutcu N Firat U Kucukoner M . Mucoepidermoid carcinoma in a breast affected by burn scars: comprehensive literature review and case report. Breast care (Basel, Switzerland). (2011) 6:293–7. doi: 10.1159/000331316 PMC3225215 22135628

[22] Yamamoto T Nakajima T Suzuki H Tagawa T Iwata T Mizobuchi T . Surgical treatment of mucoepidermoid carcinoma of the lung: 20 years’ experience. Asian Cardiovasc Thorac Ann. (2016) 24:257–61. doi: 10.1177/0218492316630494 26847635

[23] Zhang XP Hu PZ Shen SS Li XY . Clinical characteristics and prognostic analyses of 87 patients with pulmonary mucoepidermoid carcinoma. Zhonghua Zhong Liu Za Zhi. (2018) 40:452–5. doi: 10.3760/cma.j.issn.0253-3766.2018.06.010 29936772

[24] Han S-W Kim H-P Jeon PK Oh D-Y Lee S-H Kim D-W . Mucoepidermoid carcinoma of lung: potential target of EGFR-directed treatment. Lung cancer (Amsterdam, Netherlands). (2008) 61:30–4. doi: 10.1016/j.lungcan.2007.11.014 18192072

[25] Shilo K Foss RD Franks TJ DePeralta-Venturina M Travis WD . Pulmonary mucoepidermoid carcinoma with prominent tumor-associated lymphoid proliferation. Am J Surg Pathol. (2005) 29:407–11. doi: 10.1097/01.pas.0000151616.14598.e7 15725811

[26] Kalhor N Moran CA . Pulmonary mucoepidermoid carcinoma: diagnosis and treatment. Expert Rev Respir Med. (2018) 12:249–55. doi: 10.1080/17476348.2018.1428563 29338644

[27] Wang L Cheng D Wang H Cheng L Zhang X . A high-grade breast mucoepidermoid carcinoma without MAML2 rearrangement: A case report and literature review. Medicine. (2024) 103(8):e37163. doi:10.1097/MD.0000000000037163 38394503 PMC11309622

[28] Mura MD Clement C Foschini MP Vander Borght S Waumans L Hauben E . High-grade HER2-positive mucoepidermoid carcinoma of the breast: a case report and review of the literature. J Med Case Rep. (2023) 17(1):527. doi:10.1186/s13256-023-04233-0 38062474 PMC10704702

[29] Bak S Choi HY Lee JH Na JB Choi DS Cho JM . Mucoepidermoid carcinoma of the breast: A case report and literature review focused on radiological findings. Medicine. (2022) 101(26):e29745. doi:10.1097/MD.0000000000029745 35777033 PMC9239627

[30] Ye RP Liao YH Xia T Kuang R Long HA Xiao XL . Breast mucoepidermoid carcinoma: a case report and review of literature. Int J Clin Exp Pathol. (2020) 13(12):3192-9 PMC7791392 33425121

[31] Yan M Gilmore H Harbhajanka A . Mucoepidermoid Carcinoma of the Breast With MAML2 Rearrangement: A Case Report and Literature Review. Int J Clin Exp Pathol. (2020) 28(7):787–92. doi:10.1177/1066896920916779 32362174

[32] Burghel GJ Abu-Dayyeh I Babouq N Wallace A Abdelnour A . Mutational screen of a panel of tumor genes in a case report of mucoepidermoid carcinoma of the breast from Jordan. Breast J. (2018) 24(6):1102–4. doi:10.1111/tbj.13142 30251446

[33] Cheng M Geng C Tang T Song Z . Mucoepidermoid carcinoma of the breast: Four case reports and review of the literature. Medicine. (2017) 96(51):e9385. doi:10.1097/MD.0000000000009385 29390541 PMC5758243

[34] Fujino M Mori D Akashi M . Mucoepidermoid Carcinoma of the Breast Found during Treatment of Lymphoma. Case Rep Oncol. (2016) 9(3):806-14. doi:10.1159/000452792 PMC5216231 28101030

[35] Palermo MH Pinto MB Zanetti JS Ribeiro-Silva A . Primary mucoepidermoid carcinoma of the breast: a case report with immunohistochemical analysis and comparison with salivary gland mucoepidermoid carcinomas. Pol J Pathol. (2013) 3:210-5. doi:10.5114/pjp.2013.38141 24166608

[36] Turk E Karagulle E Erinanc OH Soy EA Moray G . Mucoepidermoid carcinoma of the breast. Breast J. (2013) 19(2):206–8. doi:10.1111/tbj.12080 23294278

[37] Camelo-Piragua SI Habib C Kanumuri P Lago CE Mason HS Otis CN . Mucoepidermoid carcinoma of the breast shares cytogenetic abnormality with mucoepidermoid carcinoma of the salivary gland: a case report with molecular analysis and review of the literature. Human pathology. (2009) 40(6):887–92. doi:10.1016/j.humpath.2008.11.004 19200580

[38] Hornychová H Ryska A Betlach J Bohác R Cízek T Tomsová M Obermannová R . Mucoepidermoid carcinoma of the breast. Neoplasma. (2007) 54(2):168–72 17319792

[39] Gómez-Aracil V Mayayo Artal E Azua-Romeo J Mayayo Alvira R Azúa-Blanco J Arraiza Goicoechea A . Fine needle aspiration cytology of high grade mucoepidermoid carcinoma of the breast: a case report. Acta cytologica. (2006) 50(3):344–8. doi:10.1159/000325967 16780034

[40] Di Tommaso L Foschini MP Ragazzini T Magrini E Fornelli A Ellis IO . Mucoepidermoid carcinoma of the breast. Virchows Archiv. (2004) 444(1):13-9. doi:10.1007/s00428-003-0923-y 14634807

[41] Terzi A Saglam A Uner A . A 79 year-old woman with a mass in the right breast. Turk J Cancer. (2004) 34:38-9

[42] Tjalma WA Verslegers IO De Loecker PA Van Marck EA . Low and high grade mucoepidermoid carcinomas of the breast. Eur J Gynaecol Oncol. (2002) 23(5):423–5 12440816

[43] Berry MG Caldwell C Carpenter R . Mucoepidermoid carcinoma of the breast: a case report and review of the literature. Eur J Surg Oncol. (1998) 24(1):78-80. doi:10.1016/s0748-7983(98)80135-2 9542524

[44] Markopoulos C Gogas H Livaditou A Floros D . Mucoepidermoid carcinoma of the breast. Eur J Gynaecol Oncol. (1998) 19(3):291–3 9641234

[45] Chang LC Lee N Lee CT Huang JS . High-grade mucoepidermoid carcinoma of the breast: case report. Changgeng yi xue za zhi. (1998) 21(3):352-7 9849021

[46] Lüchtrath H Moll R . Mucoepidermoid mammary carcinoma. Immunohistochemical and biochemical analyses of intermediate filaments. Virchows Arch A Pathol Anat Histopathol. (1989) 416(2):105-13. doi:10.1007/BF01606314 2480681

[47] Pettinato G Insabato L De Chiara A Manco A Petrella G . High-grade mucoepidermoid carcinoma of the breast. Fine needle aspiration cytology and clinicopathologic study of a case. Acta cytologica. (1989) 33(2):195–200. 2929221

[48] Hanna W Kahn HJ . Ultrastructural and immunohistochemical characteristics of mucoepidermoid carcinoma of the breast. Human pathology. (1985) 16(9):941–6. doi:10.1016/s0046-8177(85)80133-7 4029947

[49] Hastrup N Sehested M . High-grade mucoepidermoid carcinoma of the breast. Histopathology. (1985) 9(8):887–92. doi:10.1111/j.1365-2559.1985.tb02873.x 4054847

[50] Leong AS Williams JA . Mucoepidermoid carcinoma of the breast: high grade variant. Pathology. (1985) 17(3):516–21. doi:10.3109/00313028509105513 4069771

[51] Ratanarapee S Prinyar-Nussorn N Chantarakul N Pacharee P . High-grade mucoepidermoid carcinoma of the breast. A case report. J Med Assoc Thai. (1983) 66(10):642–8. 6663220

[52] Fisher ER Palekar AS Gregorio RM Paulson JD . Mucoepidermoid and squamous cell carcinomas of breast with reference to squamous metaplasia and giant cell tumors. Am J Surg Pathol. (1983) 7(1):15–27. doi:10.1097/00000478-198301000-00002 6829846

[53] Kovi J Duong HD Leffall LS Jr . High-grade mucoepidermoid carcinoma of the breast. Arch Pathol Lab Med. (1981) 105(11):612–614. 6895301

[54] Chen Y Zhu C Xu P Yao J . A case report of pancreatic mucoepidermoid carcinoma responded to gemcitabine and paclitaxel. Heliyon. (2024) 10(10):e31673. doi: 10.1016/j.heliyon.2024.e31673 38831837 PMC11145544

[55] Costa MR Sugita DM Vilela MH da Silva Mendonça RP de Morais DT Júnior PC . Mucoepidermoid carcinoma of the penis: case report and literature review. Can Urol Assoc J. 9(1–2):E27–9. doi: 10.5489/cuaj.2126 PMC4301965 25624963

[56] Khataniar H Senthil S Deep SS Ramesh R Y K I . Intraosseous Mucoepidermoid Carcinoma of the anterior mandible: A Case Report. Cureus. 14(5):e25036. doi: 10.7759/cureus.25036 PMC9198741 35719820

[57] Janakiram TN Sagar S Sharma SB Subramaniam V . Primary Mucoepidermoid Carcinoma of the Lacrimal Sac - a Case Report and Literature Review. Klinicka onkologie : casopis Ceske a Slovenske onkologicke spolecnosti. 29(4):1–4. doi: 10.14735/amko2016291 27534787

[58] Zhang HY Yang HY . Mucoepidermoid carcinoma in the infratemporal fossa: A case report. World J Clin Cases. 8(14):3090–6. doi: 10.12998/wjcc.v8.i14.3090 PMC7385606 32775391

[59] Mura MD Clement C Foschini MP Vander Borght S Waumans L Van Eyken P . High-grade HER2-positive mucoepidermoid carcinoma of the breast: a case report and review of the literature. J Med Case Rep. 17(1):527. doi: 10.1186/s13256-023-04233-0 PMC10704702 38062474

[60] Fois AG Diana G Arcadu A Maras V Crivelli P Putzu C . Bronchial mucoepidermoid carcinoma: A case report. Int J Surg Case Rep. 31:159–62. doi: 10.1016/j.ijscr.2017.01.042 PMC5288321 28152492

[61] Li Z Nguyen Canh H Nguyen Thi K Takahashi K Nguyen Thi Q Le Thanh D . Primary hepatobiliary mucoepidermoid carcinoma: a case report and review of literature. Med Mol Morphol. 57(3):233–43. doi: 10.1007/s00795-024-00390-3 38904830

[62] Yamamoto M Hirata K Tuneyoshi M Yoshida Y Matsuda H Gion T . Mucoepidermoid carcinoma of the anal canal: A case report and review of the literature. Mol Clin Oncol. 9(5):504–6. doi: 10.3892/mco.2018.1706 PMC6174425 30345043

